# Expression Profiling of *Castanea* Genes during Resistant and Susceptible Interactions with the Oomycete Pathogen *Phytophthora cinnamomi* Reveal Possible Mechanisms of Immunity

**DOI:** 10.3389/fpls.2017.00515

**Published:** 2017-04-11

**Authors:** Carmen Santos, Sofia Duarte, Sara Tedesco, Pedro Fevereiro, Rita L. Costa

**Affiliations:** ^1^Molecular Biology Lab, Instituto Nacional de Investigação Agrária e Veterinária, I.P.Oeiras, Portugal; ^2^Plant Cell Biotechnology Lab, Instituto de Tecnologia Química e Biológica António Xavier (Green-it Unit), Universidade Nova de LisboaOeiras, Portugal; ^3^Departamento Biologia Vegetal, Faculdade de Ciências da Universidade de LisboaCampo Grande, Portugal; ^4^Centro de Estudos Florestais, Instituto Superior de Agronomia, Universidade de Lisboa - Tapada da AjudaLisboa, Portugal

**Keywords:** *Castanea*, *Phytophthora cinnamomi*, ink disease, plant biotic interactions, digital PCR

## Abstract

The most dangerous pathogen affecting the production of chestnuts is *Phytophthora cinnamomi* a hemibiotrophic that causes root rot, also known as ink disease. Little information has been acquired in chestnut on the molecular defense strategies against this pathogen. The expression of eight candidate genes potentially involved in the defense to *P. cinnamomi* was quantified by digital PCR in *Castanea* genotypes showing different susceptibility to the pathogen. Seven of the eight candidate genes displayed differentially expressed levels depending on genotype and time-point after inoculation. *Cast_Gnk2-like* revealed to be the most expressed gene across all experiments and the one that best discriminates between susceptible and resistant genotypes. Our data suggest that the pre-formed defenses are crucial for the resistance of *C. crenata* to *P. cinnamomi*. A lower and delayed expression of the eight studied genes was found in the susceptible *Castanea sativa*, which may be related with the establishment and spread of the disease in this species. A working model integrating the obtained results is presented.

## Introduction

The European chestnut tree (*Castanea sativa* Mill.), also known as sweet chestnut, is a species of flowering tree of the Fagaceae family, native to Europe and Asia Minor and widely cultivated throughout the temperate world. In the Mediterranean region, the European chestnut has a significant economic role mainly because of the high quality of its nuts, which production is about 117,207 tons per year (FAOSTAT, 2016, faostat.fao.org).

The ubiquitous hemibiotrophic oomycete *Phytophthora cinnamomi* is the most severe pathogen affecting European chestnut, causing root rot and death, resulting in large losses in chestnut production. In Portugal, there was a decrease of 27.3% in the distribution area of chestnut between 2002 and 2004, due to *P. cinnamomi* infections (Martins et al., 2007). *P. cinnamomi* has an exceptionally wide host range, being able to destroy thousands of plant species worldwide and causing devastating impacts in natural ecosystems, agriculture, horticulture, forestry and in the nursery industry (Hardham, 2005; Cahill et al., 2008; Robin et al., 2012; Kamoun et al., 2014). Among chestnuts, the Japanese chestnut (*Castanea crenata* Sieb. et Zucc) and the Chinese chestnut (*Castanea mollissima* Bl.) show resistance to *P. cinnamomi* (Crandall et al., 1945). Therefore, these East Asian species have been used in chestnut breeding programs as donors of resistance to root rot in Europe since the last century.

Plants developed diverse constitutive and inducible defense mechanisms against pathogens. Three different defense layers have been recognized (Freeman and Beattie, 2008). In the first layer, pre-existing mechanisms comprise the first line of immune defense and include physic-chemical barriers, such as waxy cuticular layers, cell wall and antimicrobial compound (Doughari, 2015). The second and third layers are inducible: the PAMP-triggered immunity (PTI) layer that relies on the recognition of pathogen-associated molecular patterns (PAMPs) by pattern recognition receptor (PRR), activating early resistance responses, such as transcriptional reprograming; and the effector-triggered immunity (ETI) layer, which is elicited by pathogen effectors and activates host resistance genes that usually results in hypersensitive response (HR) mediated by salicylic acid (SA) signaling (Jones and Dangl, 2006; Zhang et al., 2013; Cui et al., 2015).

Plant defense mechanisms against *Phytophthora* have been studied in different species at the histological, physiological, biochemical, and molecular levels (recently reviewed in Fawke et al., 2015 and Oßwald et al., 2014). These authors report that all three layers of defense against *Phytophthora* are active in the hosts. In particular, authors mention as part of defense mechanisms the presence of genes involved in oxidative stress (e.g., peroxidases), SA-responsive genes (mainly pathogenesis related proteins), resistance genes involved in effectors recognition (TIR-NBS-LRR) and membrane associated transcription factors (NAC family). In Fagaceae, *P. cinnamomi*-*Quercus suber* interactions have been studied at the transcriptomic level, and a hypothetical molecular mechanism model has been proposed where only ETI is described (Coelho et al., 2011).

Ten years ago, a breeding program was initiated in Portugal to introgress resistance genes of Asian species (*C. mollissima* and *C. crenata*) into *C. sativa*, by controlled crosses (Costa et al., 2011). Nevertheless, the knowledge about the molecular mechanism driving chestnut resistance to the ink disease, caused by *P. cinnamomi* is still scarce. To overcome such limitation, a study has been conducted to identify candidate genes differentially expressed in roots of the susceptible species, *C. sativa*, and the resistant one, *C. crenata*, observed after *P. cinnamomi* inoculation (Serrazina et al., 2015). In this work pools of RNA from 2, 4, and 7 days of inoculated and non-inoculated roots of the two species were sequenced using a Roche 454 platform. Upon infection, Japanese chestnut up regulated twice the number of differentially expressed genes when compared with the susceptible European chestnut. Differential expression analysis revealed that in *C. crenata* genes related to response to biotic stresses were more expressed than in *C. sativa*. After *P. cinnamomi* inoculation, the differential expressed genes identified between both species were involved in recognition of pathogen attack, regulation of plant immune response, stress adaptation and recovery. While this approach constituted a valuable contribution to the *Castanea* genomic resources, more precise studies are required to validate the candidate genes identified and to understand the molecular mechanisms of resistance to *P. cinnamomi* in the *Castanea* genus.

The aim of this study is to evaluate the early expression of candidate resistance genes to *P. cinnamomi* infection (0, 24, and 48 h) in *C. sativa* and a *C. crenata*, as well as in four hybrids (three *C. sativa* × *C. crenata* genotypes and a *C. sativa* × *C. mollissima*) with different responses to *P. cinnamomi*, produced by the Portuguese chestnut breeding program and to add to the understanding of the molecular mechanisms of resistance to this pathogen in the *Castanea* genus.

Among the different methods available to quantify gene expression in plants, digital PCR (dPCR) is emerging as an absolute quantification method with high precision, sensitivity and specificity (Majumdar et al., 2015). This new technology has been mainly used for biomedicine research (Kinz et al., 2015; Salvi et al., 2015; Sefrioui et al., 2015; Stabley et al., 2015). However, some studies in plant science using dPCR have also been recently released (Bahder et al., 2016; Ge et al., 2016; Kadam et al., 2016; Stevanato and Biscarini, 2016).

## Materials and methods

### Plant material and *P. cinnamomi* inoculation

Six chestnut genotypes showing different levels of resistance after inoculation with the pathogen were used in this work. In Table 1 a characterization of the resistance levels of each genotype is provided. *C. crenata* (resistant) and *C. sativa* (susceptible) genotypes were provided by TRAGSA nursery (Grupo TRAGSA-SEPI, Maceda, Spain) and correspond to the genotypes used by Serrazina et al. (2015) for root transcriptomes sequencing. Four hybrid genotypes with different responses to *P. cinnamomi* were selected from the on-going chestnut breeding program (Santos et al., 2015): three *C. sativa* × *C. crenata* hybrids (SC55, SC914 and SC903) and a *C. sativa* × *C. mollissima* hybrid (SM904), selected as a resistance control.

**Table 1:** **Characterization of six chestnut genotypes showing different levels of resistance after inoculation with ***P. cinnamomi*****.

**Sample Name**	**Species**	**Origin**	**Survival's percentage**	**Days of survival (average)**	**Level of resistance**
*C. sativa*	*Castanea sativa*	TRAGSA, Spain	0	7	Susceptible
*C. crenata*	*Castanea crenata*	TRAGSA, Spain	83	–	Resistant
SM904	*C. sativa × C. mollissima* (F1)	Portugal	46	–	Resistant
SC55	*C. sativa × C. crenata* (F1)	Portugal	38	–	Resistant
SC914	*C. sativa × C. crenata* (F1)	Portugal	0	29	Intermediate
SC903	*C. sativa × C. crenata* (F1)	Portugal	0	10	Susceptible

*Phenotypic data is presented as percentage of survival and days of survival after inoculation. Hybrid phenotyping data was assessed in Santos et al. (2015). Genotype SC903 is the most susceptible hybrid and genotype SM904 the most resistant hybrid to P. cinnamomi inoculation*.

All plant material used in this study was multiplied by *in vitro* propagation (Supplementary Figure 1). First, individual shoots from mother trees were established and multiplied on Murashige and Skoog medium (half concentration of NH_4_NO_3_ and KNO_3_), supplemented with 1 g/L and 0.1 g/L benzylaminopurine, respectively, 30 g/L sucrose and 8 g/L phyto-agar. Elongated shoots were transferred to Murashige and Skoog medium described above (without phyto-hormones) plus 3 g/L charcoal for 7–10 days. Rooting phase consists on dipping elongated shoots into 1 g/L indolebutyric acid for 1 min and then placed at a wet porous substrate, perlite:vermiculite (1:1), for 3 weeks. Rooted plants are transferred to pots with peat:vermiculite:perlite (1:1:1). All propagation steps were performed under controlled conditions with temperatures ranging between 18 and 24°C, photoperiod 16 h light/8 h dark.

*P. cinnamomi* root inoculation was performed 80 days after plant acclimatization under controlled conditions and according to Santos et al. (2015). Briefly, *P. cinnamomi* inoculum was prepared by growing mycelia on sterilized vermiculite, which were thoroughly moistened with a solution of 200 mL V8 vegetable juice, 3 g of calcium carbonate and 800 mL distilled water. Afterwards, this mixture was incubated for 3 weeks in darkness at 25°C. Inoculum was placed into the substrate of each pot at a concentration of 5% (v/v), minimizing root disturbance, and flooded for 1 h to stimulate zoospore release, promoting the root infection and disease development. Aiming cover diverse facets of host defense response, three root biological replicates were harvested per genotype at 0 (uninfected), 24 and 48 hours post inoculation (hpi), corresponding to different stages of pathogen colonization (Redondo et al., 2015). Roots were gently washed and separated from the aerial part, frozen in liquid nitrogen and stored at −80°C until RNA isolation.

### Selection of candidate genes

Genes were selected from the 283 *C. crenata* differentially expressed genes (DEGs), previously identified by Serrazina et al. (2015). Transcriptomic data sets are publicly available on the Hardwood Genomics Project website (http://hardwoodgenomics.org/) and in the Short Read Archive at NCBI (http://www.ncbi.nlm.nih.gov/) with the reference PRJNA215368. In this study, gene selection parameters were: (1) DEGs with the log_2_ of the ratio between *C. crenata* inoculated (*Cci*) and non-inoculated (*Ccn*) reads higher than 1.5 (Log_2_*Cci/Ccn* >1.5); (2) The correspondent DEGs in *C. sativa* transcriptomes with Log_2_*Csi/Csn* <1.5 or absent; (3) DEGs not involved in general biological processes, such as oxidative, metabolic and transporter activities; (4) DEGs involved in defense response and categorized in pathogen recognition which usually triggers resistance signaling pathways, anti-pathogen proteins, cell wall modification proteins and transcription factors involved in the regulation of other defense related processes.

### Primer and probe design

Primers and TaqMan®-Probes sequences were designed using Primer 3 software version 0.4.0 (available at http://bioinfo.ut.ee/primer3-0.4.0/primer3/) and were synthesized by Life Technologies. Conserved domain sequences were avoided to primer design in order to increase the specificity. Primer selection parameters were set: primer size of 18–20 bp, a product size range of 100–150 bp; a primer melting temperature of 58–60°C; primer GC content of 30–60%, primer with no more than two G/C in the last five 3′ end nucleotides and no more than three G's runs within the sequences. TaqMan®-Probes design followed the same criteria, except size between 18 and 30 bp and melting temperatures ranging 68–70°C. Probes were labeled with FAM or VIC dye on the 5′ end and NFQ (Non-fluorescent Quencher) on the 3′ end.

### RNA isolation and cDNA synthesis

Total RNA from root tissue was isolated as described in le Provost et al. (2007), without DNase treatment. mRNA was purified using the Dynabeads® mRNA Purification Kit (Life Technologies) using half volume of dynabeads and buffers and according to the manufacturer's instructions. RNA and mRNA quality was assessed by measuring the ratios of absorbance at 260/280 and 230/280 using a nanodrop; the results obtained were, in average, absorbance_260/280_ = 1.92 and absorbance_230/280_ = 1.77. mRNA was used for cDNA synthesis using RevertAid H Minus Reverse Transcriptase kit (ThermoFisher Scientific). 0.5 μg of oligo(dT)_18_ primer and DEPC-treated water to make 12.5 μl were added to 50 ng of mRNA and incubated at 65°C for 5 min. Then, 1x reaction buffer [250 mM Tris-HCl (pH 8.3 at 25°C), 250 mM KCl, 20 mM MgCl_2_, 50 mM DTT], 20 units of ribolock RNase inhibitor, dNTP Mix (1 mM final concentration) and 200 units of RevertAid H Minus Reverse Transcriptase were added to the previous mixture and incubated 60 min at 42°C. Reverse transcriptase was inactivated by heating at 70°C for 10 min.

### QuantStudio™ 3D digital PCR

QS3D digital PCR System (Life Technologies) was used to quantify gene expression of eight *P. cinnamomi* resistance candidate genes in the roots of the six chestnut genotypes under study. 0.125 to 2.5 ng of cDNA and two TaqMan® probes (specific primers/probe mix) one labeled with FAM and the other with VIC were added to the QS3D master mix. Each QS3D chip was loaded with 14.5 μL reaction and sealed, using an automatic chip loader (Life Technologies) according to the manufacturer's instructions. The QS3D chip amplification was performed on the dual flat-block GeneAmp® PCR System 9700 thermal cycler with the following conditions: 96°C 10 min, 60°C 2 min and 98°C 30 s for 40 cycles, then 60°C for 2 min and hold at 25°C (avoiding chip condensation). After amplification, the chips were imaged on the QS3D Instrument, which assesses raw data and calculates the estimated concentration of the nucleic acid sequence targeted by FAM and VIC labeled probes assuming a Poisson distribution (Fazekas de St Groth, 1982). Data analysis and management were performed using QuantStudio™ 3D Analysis Suite™ software (https://apps.lifetechnologies.com/quantstudio3d/). Chip quality control was calculated based on the number of partitions that exceed the selected quality threshold (fixed automatically at 0.5) on the total number of wells filled correctly. The software automatically removed data points that did not meet the default quality threshold. Cn/μL were calculated by software taking into account the dilution factor.

To estimate the absolute copies of template molecules, present in the sample volume, the software applies a quantification algorithm based on the Poisson model. The estimated Cn/μL mean values are presented in a confidence interval at 95%. Standard deviation was calculated assuming the Poisson distribution of the data. Shapiro-Wilk test was used to confirm the type of data distribution. Comparison of gene expression between *C. sativa* and each of the other genotypes was done using the Wilcoxon-Mann-Whitney (non-parametric) test.

## Results

### *P. cinnamomi* phenotyping

The hybrid genotypes used in this study were previously phenotyped to *P. cinnamomi* susceptibility after root inoculation (Santos et al., 2015). Results obtained are consistent with those previously published. Forty-six percent of SM904 plants survived inoculation, this being the most resistant hybrid under study. About 38% of SC55 plants also survived to the inoculation, representing the most resistant hybrid of *C. sativa* × *C. crenata* crosses. On the other hand, none of the SC914 and SC903 plants survived to the inoculation; in these cases, the average of days of survival were used to discriminate their level of response (Table 1). Response to *P. cinnamomi* was also evaluated for *C. sativa* and *C. crenata* genotypes showing contrasting responses: *C*. *sativa* plants died 1 week after inoculation, while 83% of *C. crenata* plants survived to inoculation (Table 1).

### Resistance candidate genes to *P. cinnamomi*

Using the gene selection parameters defined, eight candidate genes were identified (Table 2). These genes codify proteins potentially involved in the three layers of defense to *P. cinnamomi* infection, previously described (Freeman and Beattie, 2008) two pathogen recognition proteins (*Cast_LRR-RLK* and *Cast_C2CD*) which trigger resistance signaling pathways; three transcription factors (*Cast_WRKY 31, Cast_ABR1* and *Cast_Myb4*) involved in the regulation of other defense processes; a ubiquitination regulator (*Cast_RNF5*); a cell wall modification enzyme (*Cast_PE-2*) and an antifungal protein (*Cast_Gnk2-like*). All genes selected were up-regulated after inoculation in *C. crenata* root transcriptomes (Serrazina et al., 2015).

**Table 2:** **Candidate genes identification**.

**Gene acronyms**	**Log_2_(*Cci/Ccn)***	***P-value***	**BLAST best hit (*Species*)**
*Cast_Gnk2-like*	2.88	1.13e^−12^	Gnk2-homologous domain, Cysteine-rich repeat secretory protein 38 (*Oryza sativa*)
*Cast_PE-2*	2.98	4.90e^−08^	Pectinesterase 2 (*Populus trichocarpa*)
*Cast_ABR1*	4.64	2.30e^−13^	Pathogenesis-related transcriptional factor, Ethylene-responsive transcription factor (AP2/ERF) ABR1 (*Ricinus communis*)
*Cast_C2CD*	2.48	6.60e^−05^	C2 calcium-dependent membrane targeting, C2 domain-containing protein (*Arabidopsis thaliana*)
*Cast_LRR-RLK*	2.32	6.35e^−07^	LRR receptor-like serine/threonine-protein kinase (*Ricinus communis*)
Cast_Myb4	2.95	1.28e^−08^	SANT domain, DNA binding, Myb-related protein Myb4 (*Vitis vinefera*)
Cast_WRKY 31	1.71	8.18e^−06^	WRKY transcription factor 31 (*Arabidopsis thaliana*)
Cast_RNF5	2.97	1.19e^−05^	Zinc finger, RING finger protein 5 (*Lactobacillus crispatus*)

*Log_2_ ratio between C. crenata inoculated and C. crenata non-inoculated, P-value and BLAST best hit information is available in Serrazina et al. (2015)*.

The *P. cinnamomi* resistance candidate genes, their respective *contig* name (Serrazina et al., 2015), primers and TaqMan®-Probes sequences are listed in Supplementary Table 1.

### Accuracy and precision of QS3D quantification method

QuantStudio™ 3D AnalysisSuite™ software evaluates if the data on a chip are reliable based upon loading, signal, and noise features. Quality indicators (red, yellow or green flags, corresponding from low to high quality, respectively) are displayed for each chip. As an example, the output of the chips used to quantify *Cast_WRKY 31* and *Cast_Myb4* expression in three biological replicates (1 chip per replicate) of *C. crenata* genotype, 48 hpi, are shown in Supplementary Figure 2. The continuous green color displayed in each chip confirms high quality loading (Supplementary Figure 2A). Nevertheless, some condensation occurred on the corners, presented by yellow or red data points. White dots were automatically filtered out because they did not meet the default quality threshold. A random distribution of each target gene amplified (FAM, VIC or both dyes) and negative reactions (non-amplified wells) are shown (Supplementary Figures 2B,C). Clustering of the scatter plots of the biological triplicates allowed verifying the technical homogeneity of the results. The dilution factor was considered by the software to calculate the number of counts per microliter (Cn/μL).

### Chestnut gene expression profiling

**Figure 1:**
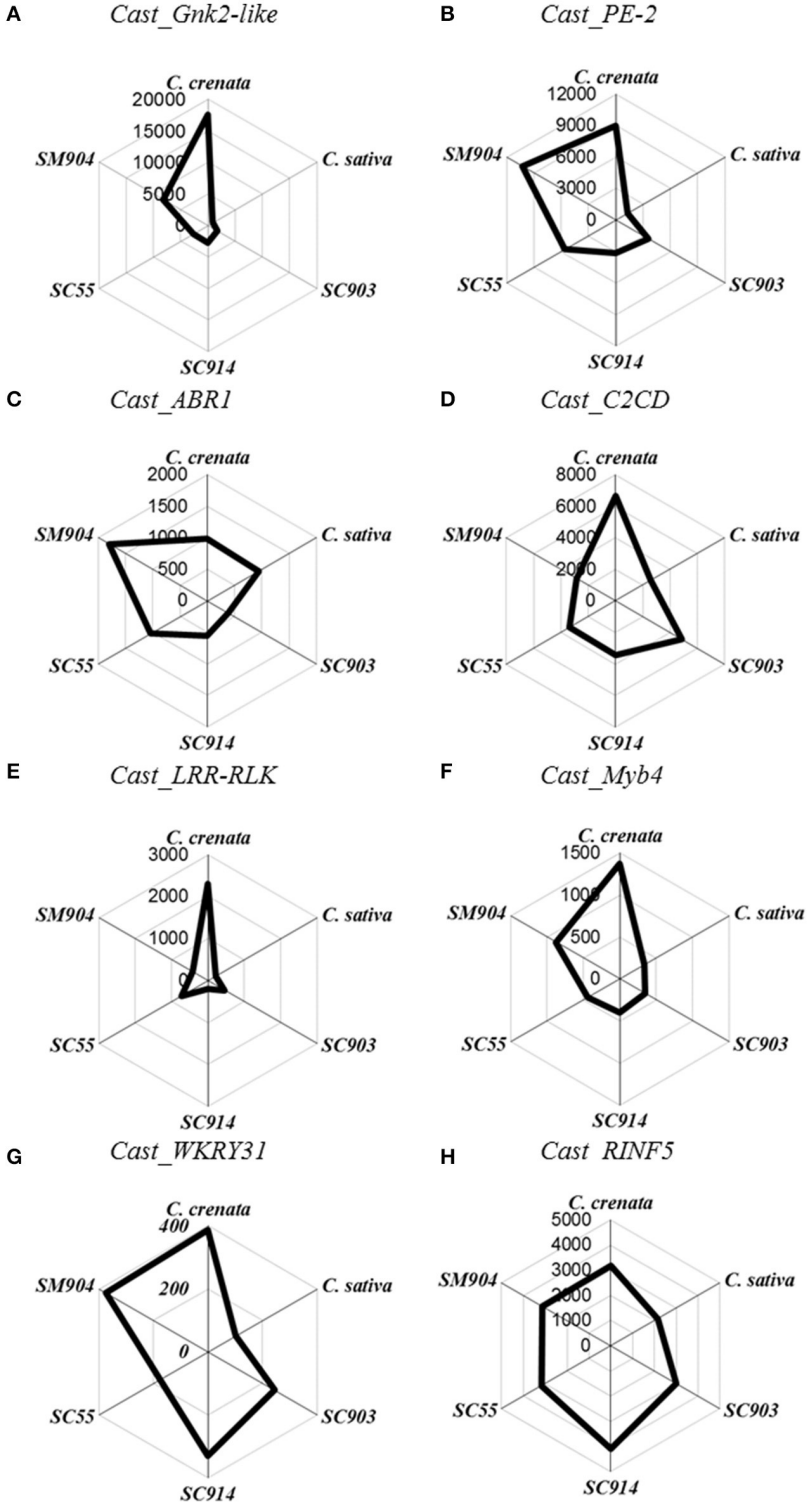
**Radar plots of copy number/μL of the eight genes in non-inoculated roots**. Starting on top and following clockwise, *C. crenata, C. sativa*, SC903, SC914, SC55, and SM904. **(A)**, *Cast_Gnk2-*like; **(B)**, *Cast_PE-2*; **(C)**, *Cast_ABR1*; **(D)**, *Cast_C2CD*; **(E)**, *Cast_LRR-RLK*; **(F)**, *Cast_Myb4*; **(G)**, *Cast_WRKY31*, and **(H)**, *Cast_RNF5*.

**Figure 2:**
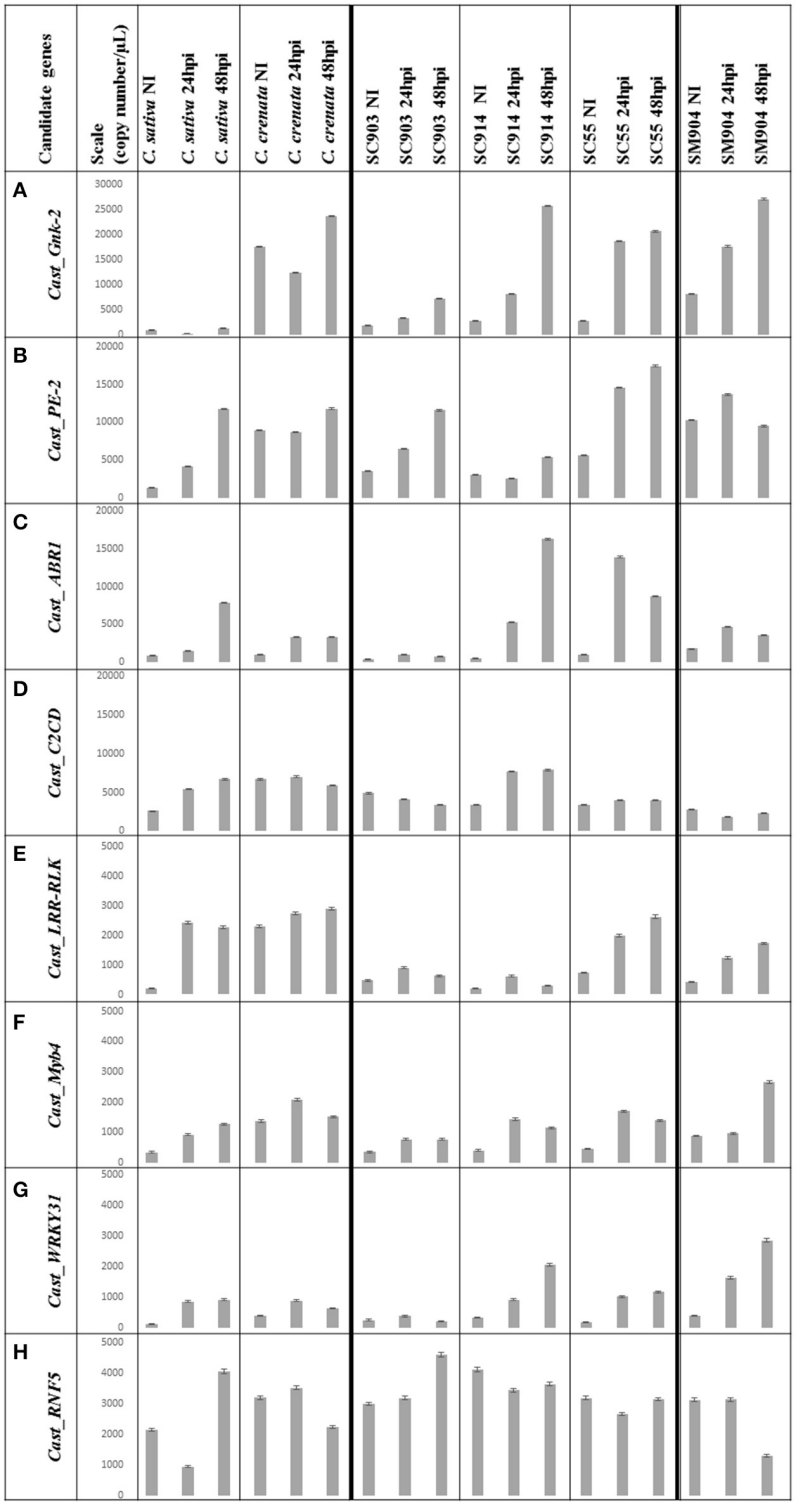
**Copy number (Cn)/μL variation of the eight genes under study**. Data is presented from *C. sativa* and *C. crenata*, from the *C. sativa* × *C. crenata* hybrids, from the most susceptible (SC903) to the most resistant (SC55), and from the resistant *C. sativa* × *C. mollissima* hybrid (SM904). For each genotype, Cn/μL for 0 hpi (not inoculated), 24 hpi and 48 hpi is shown. The mean value of each bar corresponds to the quantification of biological triplicates, calculated by the software assuming a Poisson distribution; error bars correspond to standard deviations. Y-axis, copy number/μL; X-axis, sample name × treatment; NI, non-inoculated; hpi, hours post-inoculation. **(A)** Scale adjusted to 30,000 copies/μL; **(B–D)**, scale adjusted to 20,000 copies/μL; **(E–H)**, scale adjusted to 5,000 copies/μL.

Transcripts copy number variation among the three time-points for six chestnut genotypes is presented in Figures 1, 2. The Cn/μL ranged from approximately 100 to 27,000, with the lowest values obtained for the expression of the *C. sativa* genotype and the most susceptible *C. sativa* × *C. crenata* hybrid (SC903) under non-inoculated conditions (Figures 1, 2). Aside from *Cast_ABR1*, transcription factors presented the lowest Cn/μL, particularly in the most susceptible genotypes and in the two first time points (non-inoculated and 24 hpi).

Before inoculation there is a clear differentiation in gene expression between *C. sativa* and *C. crenata*. Except for *Cast_ABR1* and *Cast_RINF5*, the pre-inoculated expression of all other genes is significantly higher in *C. crenata* (Figure 1). This pattern, with some variation, holds for the two most resistant hybrids tested (SM904 and SC55).

The expression profiles varied depending on genotype susceptibility, mainly for the *Cast_Gnk2-like, Cast_PE-2, Cast_LRR-RLK* and *Cast_Myb4* genes (Figures 1, 2A,B,E,F). *Cast_Gnk2-like* was revealed to be the most expressed gene, whose expression increases from the most susceptible to the most resistance genotype (Figures 1A, 2A). On the other hand, *Cast_RNF5* and *Cast_C2CD* displayed to have the less variation between samples and time points. In most cases, transcript abundance was higher in *C. crenata* than in *C. sativa*. Regardless, there is little evidence of correlation between gene expression and resistance when hybrids are considered.

In all the analyzed profiles, the expression levels of the eight candidate genes changed along the time points. Before inoculation with the pathogen, transcripts of all candidate genes accumulated to higher levels in *C. crenata* than in *C. sativa*, mainly for *Cast_Gnk2-like, Cast_PE-2, Cast_C2CD, Cast_LRR-RLK* and *Cast_Myb4* genes (Figure 1). These differences were significant (α = 0.05) between *C. sativa* and *C. crenata*, as well as, between *C. sativa* and hybrids, for all genes under study. Nevertheless, the intermediate *C. sativa* × *C. crenata* hybrid (SC914) showed similar Cn/μL with *C. sativa* in non-inoculated samples for *Cast_PE-2, Cast_C2CD* and *Cast_LRR-RLK*. Except for *Cast_Gnk2-like, Cast_C2CD* and *Cast_LRR-RLK*, resistant *C. sativa* × *C. mollissima* hybrid (SM904) showed similar expression profiles to *C. crenata*, in non-inoculation conditions.

Considering the whole experiment, there is a tendency for the higher accumulation of the transcripts after 48 hpi. However, in the majority of cases, *Cast_Myb4* is more expressed at 24 hpi than 48 hpi. This difference observed between time points decreases gradually from the resistant *C. crenata* to the susceptible genotypes, reaching the point where *C. sativa* expression is higher at 48 hpi (Figure 2F).

## Discussion

Basic knowledge on the molecular defense mechanisms against *P. cinnamomi* infection is required in both resistant and susceptible genotypes. The expression of eight resistance candidate genes was evaluated before and after inoculation of *C. sativa, C. crenata* and four interspecific hybrids of the on-going Portuguese chestnut breeding program. *C. crenata* showed the highest expression of these genes, especially under non-inoculated conditions, opposing to *C. sativa*, in which the lower transcripts abundance was measured. This is similar the observed in the analyses of the transcriptome where, except for *Cast_Myb4* and *Cast_WRKY31*, all other studied genes are expressed at higher levels prior infection in *C. crenata* vs. *C. sativa*. The results seem to show that effectiveness of the first layer of defense mechanisms may explain the difference in *P. cinnamomi* resistance between *C. sativa and C. crenata*. Resistance may have evolved during host-pathogen coevolution, since *P. cinnamomi* is native to Asia (Ko et al., 1978; Zentmyer, 1988; Zhang et al., 1994) and *C. crenata* (Japanese chestnut) seem to be the ancestral of the other species of *Castanea* genus (Lang et al., 2007). Nevertheless, the correlation between gene expression and resistance seems to be weaker for the hybrid genotypes.

### Physical and chemical barriers to *P. cinnamomi* infection

The secretion of toxic compounds is an effective defense mechanism against pathogens in plants (Wittstock and Gershenzon, 2002; Montesinos, 2007). Ginkbilobin-2 (Gnk2) is a protein secreted by *Ginkgo biloba* seeds that exhibits an antifungal activity (Wang and Ng, 2000; Sawano et al., 2007). Gnk2 has a plant-specific cysteine-rich motif DUF26 (domain of unknown function 26, also known as stress-antifungal domain: PF01657) which belongs to cysteine-rich receptor-like kinases (CRKs) (Miyakawa et al., 2014) not showing any similarity with other known antimicrobial proteins (Sawano et al., 2007; Miyakawa et al., 2014). It was recently shown that Gnk2 can also activate actin-dependent cell death (Gao et al., 2016). Therefore, Cast_Gnk2-like may prevent pathogen growth either by its chemical properties or by inducing HR-related cell death.

The highest *Cast_Gnk2-like* expression registered in non-inoculation conditions suggests that *C. crenata* root surroundings may be a hostile environment for fungal and fungal-like pathogens, such as *P. cinnamomi*. On the other hand, *C. sativa* showed a very low *Cast_Gnk2-like* expression level, even after pathogen inoculation. Considering the whole experiment, *Cast_Gnk2-like* was the most expressed gene and that best discriminates between susceptible and resistant genotypes (Figures 1A, 2A). The isolation and purification of Cast_Gnk2-like protein may have biotechnological applications, such as the development of an antimicrobial phytopharmaceutical against *P. cinnamomi*.

A crucial constitutive defense is the formation of wall appositions that comprise a physical barrier to pathogen growth (Hardham and Blackman, 2010). The reinforcement of plant cell walls by calcium-pectate gel apposition with the involvement of pectinesterases have been shown to confer resistance to *Phytophthora* species (Kieffer, 2000; Wiethölter et al., 2003). In this study, expression levels of *Cast_PE-2* show that this enzyme may have a role on *P. cinnamomi* resistance in chestnut. Compared with *C. sativa, C. crenata* exhibited higher *Cast_PE-2* expression levels in all time points, mainly in the non-inoculated samples (about 10x more), suggesting that their cell walls may be more resistant to pathogen penetration. After the first pathogen contact, *Cast_PE-2* expression increases, suggesting a possible continuing apposition of pectates in cell walls, probably to inhibit further colonization. This seems to be more important in a late stage of infection (48 hpi) except for the *C. sativa* × *C. mollissima* hybrid. Possibly, other resistance mechanisms may be activated earlier in this hybrid and control the infection.

### Pathogen recognition and successive host response regulation

Generally, during pathogen infection, PAMPs are recognized by pattern-recognition receptors (PRRs) at the plant's cell surface. The best-studied class of plant PRRs are receptor-like kinases (RLKs), which have an ectodomain of leucine-rich repeats (LRRs) involved in PAMP perception (Jones and Dangl, 2006; Boller and Felix, 2009; ten Hove et al., 2011). Resistance related LRR proteins have been shown to be differentially expressed in global transcript profiling studies in *Phytophthora* spp. infection response (Ballvora et al., 2002; van der Vossen et al., 2003; Gao et al., 2005; Boava et al., 2011; Coelho et al., 2011; Mahomed and Berg, 2011). Contrasting to *C. sativa, C. crenata* has a much higher (about 10x more) *Cast_LRR-RLK* expression before inoculation (Figures 1E, 2E), which may mediate a fast and effective response against *P. cinnamomi*, suggesting that this earlier recognition is part of the resistance phenotype. Furthermore, *Cast_LRR-RLK* expression increased after *P. cinnamomi* inoculation for all *Castanea* genotypes. Considering the previous studies on LRR biological functions in Fagaceae, *Cast_LRR-RLK* may recognize and interact with PAMPs molecules, secreted by *P. cinnamomi*, activating downstream signaling responses (Coelho et al., 2011).

RLKs have an intracellular kinase domain involved in a downstream signaling via MAPK cascades which trigger defense-related pathways by transcription factors activation (Pitzschke et al., 2009; Tena et al., 2011), such as WRKY, MYB and Ethylene-responsive transcription factors (Oñate-Sánchez and Singh, 2002; Kim and Zhang, 2004; Dubos et al., 2010). WRKY proteins regulate pathogen- and salicylic-acid (SA)-responsive genes having a pivotal role in host response to stress (Eulgem, 2000; Dong et al., 2003; Eulgem and Somssich, 2007; Yang et al., 2009; Shimono et al., 2012). In particular, the overexpression of WRKY 31 in rice seedlings after treatment with a hemibiotrophic fungus (*Magnaporthe grisea*) was associated with blockade of pathogen invasion (Zhang et al., 2008). *Cast_WRKY 31* may have a role in the response of chestnut to *P. cinnamomi* infection, since its expression increased in inoculated samples when compared with non-inoculated ones, probably regulating SA-responsive genes expression. This increase seems more consistent in the more resistant hybrids.

SA induces defense responses against biotrophic pathogens (Loake and Grant, 2007; Vlot et al., 2009). High concentrations of endogenous SA may induce HR (Mur et al., 2008). SA was not quantified in this work, but Serrazina et al. (2015) found nine differentially expressed genes between infected and non-infected *C. crenata* (*Calcium-dependent protein kinase, Patatin-05, Sulfate transporter 3.1, Ocs element-binding factor 1, Arginine decarboxylase, Probable glutathione S-transferase, Pto-interacting protein 1, Acidic endochitinase and 3-ketoacyl-CoA synthase 11*) whose expression is described to be regulated by SA.

The balance between SA and other phytohormones is increasingly recognized as central to the outcome of plant–pathogen interactions (de Torres-Zabala et al., 2009). Abcisic acid (ABA) disrupts SA-mediated response and suppresses the expression of many defense-related genes. The ethylene-responsive transcription factor ABR1 is a negative regulator of ABA signaling pathway in *Arabidopsis thaliana* (Pandey et al., 2005) and its expression allows SA and lignin accumulation (Mohr and Cahill, 2007; de Torres-Zabala et al., 2009; Boatwright and Pajerowska-Mukhtar, 2013). *Cast_ABR1* expression was triggered after *P. cinnamomi* inoculation, earlier in the more resistant genotypes, suggesting that ABA may be repressed after pathogen perception. In the resistant *C. crenata* genotype the relatively low increase of *Cast_ABR1* expression may due to the efficiency of other resistant mechanisms that avoid pathogen colonization, or by independence of ABA suppression for SA signaling activation.

Genes of the MYB transcription factor family are involved in the control of specific processes including responses to biotic stresses (Dubos et al., 2010). MYB4 has been shown to repress transcription of cinnamate 4-hydroxylase (C4H) enzyme (Hemm et al., 2001). C4H catalyze the second step of the main phenylpropanoid pathway, leading to the synthesis of lignin, pigments, and defense molecules. Inactivation of C4H allows the accumulation of SA in elicited cells (Schoch et al., 2002). The expression balance of *Cast_Myb4* in *Castanea* genotypes may regulate SA accumulation vs. synthesis of phenylpropanoids. The ratio of *Cast_Myb4* expression between 24/48 hpi decreased progressively from the resistant *C. crenata*, to *C. sativa* × *C. crenata* hybrids (the most resistant to the most susceptible) to the susceptible *C. sativa*. This indicates that SA signaling may be faster (24 hpi) in resistant genotypes than in susceptible ones. As mentioned before, elevated concentrations of endogenous SA will induce expression of *Cast_Gnk2-like* and *Cast_WRKY31*. For resistant genotypes (*C. crenata* and SC55), after a probable early induction of SA pathways, expression of *Cast_Myb4* decreases at 48 hpi, which may allow the synthesis of lignin and other defense molecules.

In addition to MAPK cascades regulation to activate transcription factors, the defense regulation could be also calcium-dependent, since intracellular calcium increases upon pathogen recognition (Ma and Berkowitz, 2007). Calcium rapid and transient bursts act as a key second messenger in cell signaling, inducing HR to prevent pathogen colonization (Lecourieux et al., 2002, 2006; Ma and Berkowitz, 2007). C2 domains are ubiquitous structural modules that act in Ca^2+^-dependent membrane binding. Several small C2 proteins in plants have been shown to be involved pathogen responses (Kim et al., 2003; Lecourieux et al., 2006; Wang et al., 2009). The expression profile of *Cast_C2 domain* is not in accordance with the resistant phenotypes. Nevertheless, the expression of *Cast_C2 domain* in *C. crenata* in non-inoculation conditions is noteworthy (Figures 1D, 2D). The role of *Cast_C2 domain* to *P. cinnamomi* infection warrants further investigation. Likewise, *Cast_RNF5* showed to have the least variation between samples and time points (Figure 2H). Possibly, *Cast_RNF5* may have a role in response to *P. cinnamomi* infection, but that transcriptional regulation is not an important component of regulation.

### Hypothetical *P. cinnamomi* response mechanism in *Castanea*

**Figure 3:**
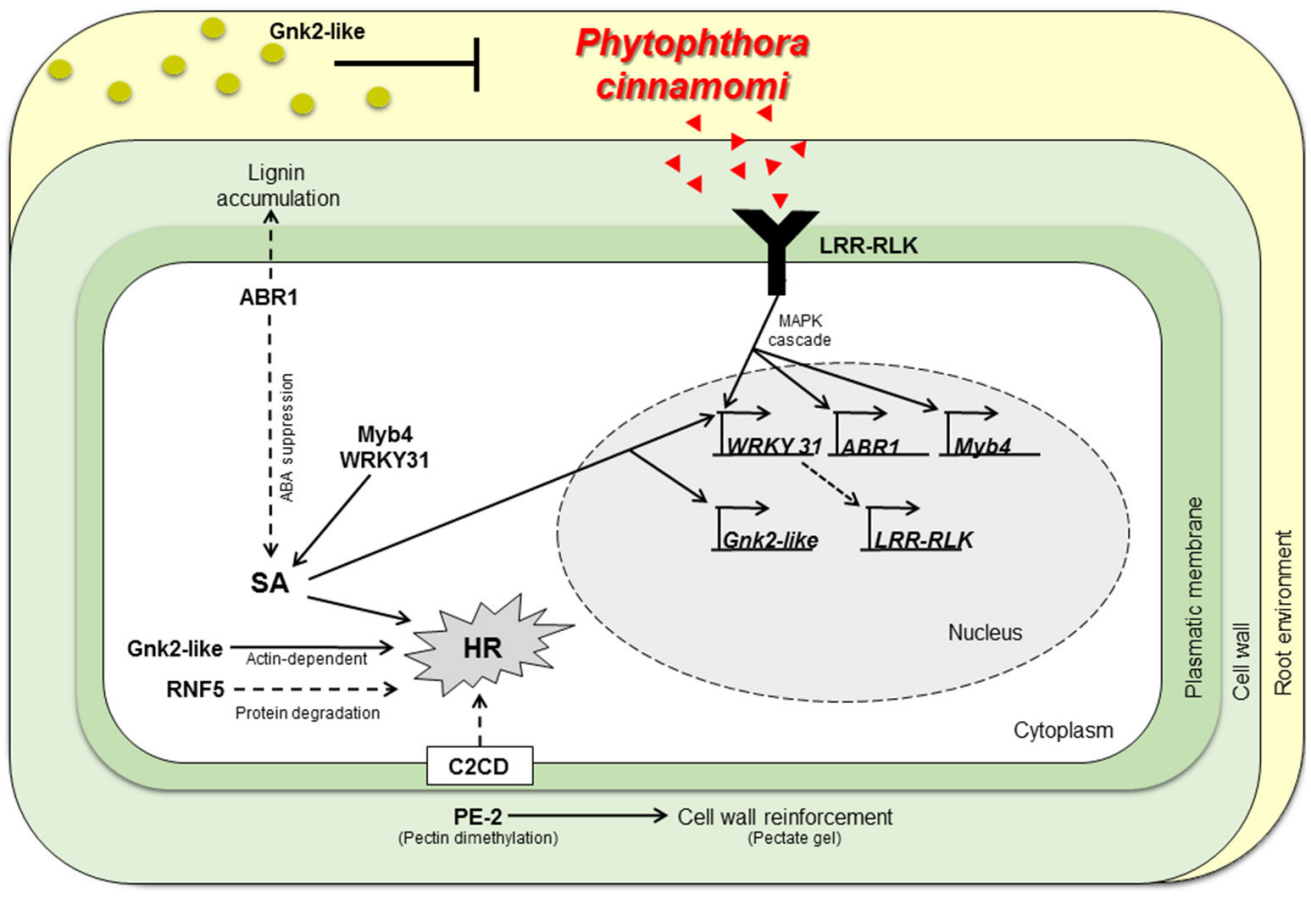
**Working model describing part of the molecular interaction of ***Castanea*** spp. to ***P. cinnamomi*** infection**. Physiochemical barriers, antifungal proteins secretion (*Cast_Gnk2-like*) and stronger cell walls (by action of *Cast_PE-2, Cast_ABR1*) respectively, may inhibit *P. cinnamomi* growth and infection. If *P. cinnamomi* overcome those barriers, specific pathogen recognition may occur, by *Cast_LRR-RLK*. Hence, host transcription is reprogramed via MAPK cascades and SA signaling. Cast_WRKY 31 should activate transcription of *LRR-RLK*. *Cast_ABR1* regulate SA accumulation via ABA suppression. HR could be activated by many mechanisms: SA or calcium signaling, via *Cast_Gnk2-like* (actin-dependent) or by vital protein degradation (by *Cast_RNF5*). Cell walls not infected may be reinforced and antifungal proteins may be secreted in more abundance, inhibiting further colonization. SA, Salicylic Acid; HR, Hypersensitive Response; Red triangles, *P*. *cinnamomi* PAMPs; Green circles, Gnk2-like proteins.

The expression profiles obtained suggest that susceptible and resistant plants may share the same response mechanisms. Despite, resistant plants show a much higher constitutive expression of the tested candidate genes before inoculation. A working model describing part of the molecular interaction of *Castanea* spp. to *P. cinnamomi* infection is presented (Figure 3): resistant genotypes present a higher expression of genes in non-inoculation conditions that may be part of a constitutive defense mechanism that prepare and protect the plant in advance to *P. cinnamomi* infection by secreting antifungal proteins and having stronger cell walls even before the contact with the pathogen. If *P. cinnamomi* overcomes those chemical and physical barriers, specific pathogen recognition proteins are earlier and more expressed in the resistant genotypes when compared to the susceptible ones. Thereafter, the transcription of the host will probably be reprogrammed via signal transduction and SA signaling. HR-related cell death is probably activated and cell walls may be reinforced in non-infected tissues, preventing further colonization.

In conclusion, the first layer of defense seems to be active and decisive in the resistance of *C. crenata* to *P. cinnamomi*. A lower and delayed expression of the eight studied genes was found in *C. sativa*, which may be related with the sensitivity of this species toward the disease. One probable explanation for this difference can be the allelic variation of the genes or gene-promoters that in *C. sativa* may condition the levels of gene expression before inoculation. *C. mollissima*, also a resistant species, may share with *C. crenata* some of the allelic variants that allow an efficient level of resistance against *P. cinnamomi*. This will be object of further research. Natural selection could have had an active role in keeping those allelic variants, since Asian species have evolved in contact with *P. cinnamomi*. This study is part of an ongoing Portuguese breeding program to introduce resistance to *P. cinnamomi in C. sativa*. This knowledge may contribute for the development of strategies to control ink disease in chestnut and other woody plants, which may include early selection of resistant genotypes.

## Author contributions

CS is the PhD student in charge of the study. She participated in the experimental design, gene selection, molecular biology, data analysis and paper writing. SD participated in the experimental design, plant material production via micropropagation, gene selection, molecular biology experiments and reviewed on successive drafts of the paper. ST participated in plant material production via micropropagation and reviewed on successive drafts of the paper. PF participated in the experimental design, interpretation of results and paper writing and reviewed on successive drafts of the paper. He supervised CS. RC is the coordinator of the project, she made the conception and design of the study, participated in writing and reviewed on successive drafts of the paper. She supervised laboratory experiments of CS, SD, and ST.

### Conflict of interest statement

The authors declare that the research was conducted in the absence of any commercial or financial relationships that could be construed as a potential conflict of interest.

## Supplementary material

Supplementary Figure 1 **Plant biological replicates of the different genotypes used in this study, obtained by ***in vitro*** culture. (A–D)** *In vitro* micropropagation phases, **(A)** Establishment of an axillary node explant from the F1 mother plant; **(B)** Shoot multiplication; **(C)** Shoot elongation; **(D)** Pre-rooting in activated charcoal medium. **(E–G)** *Ex-vitro* phases; **(E)** *Ex-vitro* rooting, **(F)** Acclimatization, plantlets primary hardening; **(G)** Plantlets with 80 days after acclimatization used for inoculation.

Supplementary Figure 2 **Representative QS3D chip views and respective plot of ***Cast_WRKY 31*** and ***Cast_Myb4*** expression for three biological replicates (***C. crenata***, 48 hpi). (A)** Chip views depicting color by quality. **(B)** Chip views depicting color by calls. **(C)** Scatter plot view from merging the three biological replicates. The data points on chip **(B)** and plot views **(C)** are color-coded according to the following fluorophores' color: FAM (blue), VIC (red), FAM + VIC (green) and not amplified (yellow). Relative intensities of FAM were plotted against VIC.

Supplementary Table 1 **Primers and probe sequences and fluorophores of eight ***Castanea crenata*** (Japanese, resistant) candidate genes to ***P. cinnamomi*** resistance**. Contig names like in (Serrazina et al., 2015)

## Abbreviations

Cn/μL: Copy number per μL
QS3D: QuantStudio™ 3D Digital PCR System
dPCR: Digital PCR
SC: *C. sativa* × *C. crenata* hybrids
SM: *C. sativa* × *C. mollissima* hybrid
HR: Hypersensitive Response
SA: Salicylic Acid.

## References

[B1] Bahder B. W. Zalom F. G. Jayanth M. Sudarshana M. R. (2016). Phylogeny of geminivirus coat protein sequences and digital PCR aid in identifying *Spissistilus festinus* as a vector of Grapevine red blotch-associated virus. Phytopathology 10, 1223–1230. 10.1094/PHYTO-03-16-0125-FI 27111804

[B2] Ballvora A. Ercolano M. R. Weiss J. Meksem K. Bormann C. A. Oberhagemann P.. (2002). The R1 gene for potato resistance to late blight (*Phytophthora infestans*) belongs to the leucine zipper/NBS/LRR class of plant resistance genes. Plant J. 30, 361–371. 10.1046/j.1365-313X.2001.01292.x 12000683

[B3] Boatwright J. L. Pajerowska-Mukhtar K. (2013). Salicylic acid: an old hormone up to new tricks. Mol. Plant Pathol. 14, 623–634. 10.1111/mpp.12035 23621321 PMC6638680

[B4] Boava L. P. Cristofani-Yaly M. Mafra V. S. Kubo K. Kishi L. T. Takita M. A.. (2011). Global gene expression of *Poncirus trifoliata, Citrus sunki* and their hybrids under infection of *Phytophthora parasitica*. BMC Genomics 12, 1–13. 10.1186/1471-2164-12-39 21241495 PMC3033816

[B5] Boller T. Felix G. (2009). A renaissance of elicitors: perception of microbe-associated molecular patterns and danger signals by pattern-recognition receptors. Annu. Rev. Plant Biol. 60, 379–406. 10.1146/annurev.arplant.57.032905.105346 19400727

[B6] Cahill D. M. Rookes J. E. Wilson B. A. Gibson L. McDougall K. L. (2008). *Phytophthora cinnamomi* and Australia's biodiversity: impacts, predictions and progress towards control. Aust. J. Bot. 56, 279–310. 10.1071/BT07159

[B7] Coelho A. C. Ebadzad G. Cravador A. (2011). Quercus suber–*P. cinnamomi* interaction: hypothetical molecular mechanism model. N. Z. J. For. Sci. 41, 143–157.

[B8] Costa R. Santos C. Tavares F. Machado H. Gomes-Laranjo J. Kubisiak T. (2011). Mapping and transcriptomic approaches implemented for understanding disease resistance to *Phytophthora cinnamomi* in *Castanea* sp. BMC Proc. 5:O18 10.1186/1753-6561-5-S7-O18

[B9] Crandall B. S. Gravatt G. F. Ryan M. M. (1945). Root disease of *Castanea* species and some coniferous and broadleaf nursery stocks, caused by *Phytophthora cinnamomi*. Phytopathology 35, 162–180.

[B10] Cui H. Tsuda K. Parker J. E. (2015). Effector-triggered immunity: from pathogen perception to robust defense. Annu. Rev. Plant Biol. 66, 487–511. 10.1146/annurev-arplant-050213-040012 25494461

[B11] de Torres-Zabala M. Bennett M. H. Truman W. H. Grant M. R. (2009). Antagonism between salicylic and abscisic acid reflects early host-pathogen conflict and moulds plant defence responses. Plant J. 59, 375–386. 10.1111/j.1365-313X.2009.03875.x 19392690

[B12] Dong J. Chen C. Chen Z. (2003). Expression profiles of the Arabidopsis WRKY gene superfamily during plant defense response. Plant Mol. Biol. 51, 21–37. 10.1023/A:1020780022549 12602888

[B13] Doughari J. (2015). An overview of plant immunity. J. Plant Pathol. Microbiol. 6, 1–11. 10.4172/2157-7471.1000322

[B14] Dubos C. Stracke R. Grotewold E. Weisshaar B. Martin C. Lepiniec L. (2010). MYB transcription factors in Arabidopsis. Trends Plant Sci. 15, 573–581. 10.1016/j.tplants.2010.06.005 20674465

[B15] Eulgem T. (2000). The WRKY superfamily of plant transcription factors. Trends Plant Sci. 5, 199–206. 10.1016/S1360-1385(00)01600-9 10785665

[B16] Eulgem T. Somssich I. (2007). Networks of WRKY transcription factors in defense signaling. Curr. Opin. Plant Biol. 10, 366–371. 10.1016/j.pbi.2007.04.020 17644023

[B17] Fawke S. Doumane M. Schornack S. (2015). Oomycete interactions with plants: infection strategies and resistance principles. Microbiol. Mol. Biol. Rev. 79, 263–280. 10.1128/MMBR.00010-15 26041933 PMC4468149

[B18] Fazekas de St Groth (1982). The evaluation of limiting dilution assays. J. Immunol. Methods 49, 11–23. 10.1016/0022-1759(82)90269-1 7040548

[B19] Freeman B. Beattie G. (2008). An overview of plant defenses against pathogens and herbivores. Plant Heal. Instr. 10.1094/PHI-I-2008-0226-01

[B20] Gao H. Narayanan N. N. Ellison L. Bhattacharyya M. K. (2005). Two classes of highly similar coiled coil-nucleotide binding-leucine rich repeat genes isolated from the Rps1-k locus encode *Phytophthora* resistance in soybean. Mol. Plant. Microbe. Interact. 18, 1035–1045. 10.1094/MPMI-18-1035 16255242

[B21] Gao N. Wadhwani P. Mühlhäuser P. Liu Q. Riemann M. Ulrich A. S.. (2016). An antifungal protein from *Ginkgo biloba* binds actin and can trigger cell death. Protoplasma 253, 1159–1174. 10.1007/s00709-015-0876-4 26315821

[B22] Ge X. Deng W. Lee Z. Z. Lopez-Ruiz F. J. Schweizer P. Ellwood S. R. (2016). Tempered *mlo* broad-spectrum resistance to barley powdery mildew in an Ethiopian landrace. Sci. Rep. 6:29558. 10.1038/srep29558 27404990 PMC4941727

[B23] Hardham A. Blackman L. (2010). Molecular cytology of *Phytophthora*-plant interactions. Australas. Plant Pathol. 39, 29–35. 10.1071/AP09062

[B24] Hardham A. R. (2005). Phytophthora cinnamomi. Mol. Plant Pathol. 6, 589–604. 10.1111/j.1364-3703.2005.00308.x 20565682

[B25] Hemm M. R. Herrmann K. M. Chapple C. (2001). AtMYB4: a transcription factor general in the battle against UV. Trends Plant Sci. 6, 135–136. 10.1016/S1360-1385(01)01915-X 11286899

[B26] Jones J. Dangl J. (2006). The plant immune system. Nature 444, 323–329. 10.1038/nature05286 17108957

[B27] Kadam S. Vuong T. D. Qiu D. Meinhardt C. G. Song L. Deshmukh R.. (2016). Genomic-assisted phylogenetic analysis and marker development for next generation soybean cyst nematode resistance breeding. Plant Sci. 242, 342–350. 10.1016/j.plantsci.2015.08.015 26566850

[B28] Kamoun S. Furzer O. Jones J. D. G. Judelson H. S. Ali G. S. Dalio R. J. D.. (2014). The Top 10 oomycete pathogens in molecular plant pathology. Mol. Plant Pathol. 16, 413–434. 10.1111/mpp.12190 25178392 PMC6638381

[B29] Kieffer F. (2000). The fungal elicitor cryptogein induces cell wall modifications on tobacco cell suspension. J. Exp. Bot. 51, 1799–1811. 10.1093/jexbot/51.352.1799 11113159

[B30] Kim C. Y. Koo Y. D. Jin J. B. Moon B. C. Kang C. H. Kim S. T.. (2003). Rice C2-domain proteins are induced and translocated to the plasma membrane in response to a fungal elicitor. Biochemistry 42, 11625–11633. 10.1021/bi034576n 14529272

[B31] Kim C. Y. Zhang S. (2004). Activation of a mitogen-activated protein kinase cascade induces WRKY family of transcription factors and defense genes in tobacco. Plant J. 38, 142–151. 10.1111/j.1365-313X.2004.02033.x 15053767

[B32] Kinz E. Leiherer A. Lang A. H. Drexel H. Muendlein A. (2015). Accurate quantitation of JAK2 V617F allele burden by array-based digital PCR. Int. J. Lab. Hematol. 37, 217–224. 10.1111/ijlh.12269 24963593

[B33] Ko W. H. Chang H. S. Su H. J. (1978). Isolates of *Phytophthora cinnamomi* from Taiwan as evidence for an Asian origin of the species. Trans. Br. Mycol. Soc. 71, 496–499. 10.1016/S0007-1536(78)80080-1

[B34] Lang P. Dane F. Kubisiak T. Huang H. (2007). Molecular evidence for an Asian origin and a unique westward migration of species in the genus *Castanea* via Europe to North America. Mol. Phylogenet. 43, 49–59. 10.1016/j.ympev.2006.07.022 17098448

[B35] Lecourieux D. Mazars C. Pauly N. Ranjeva R. Pugin A. (2002). Analysis and effects of cytosolic free calcium increases in response to elicitors in *Nicotiana plumbaginifolia* cells. Plant Cell 14, 2627–2641. 10.1105/tpc.005579 12368509 PMC151240

[B36] Lecourieux D. Ranjeva R. Pugin A. (2006). Calcium in plant defence-signalling pathways. New Phytol. 171, 249–269. 10.1111/j.1469-8137.2006.01777.x 16866934

[B37] le Provost G. Herrera R. Paiva J. Chaumeil P. Salin F. Plomion C. (2007). A micromethod for high throughput RNA extraction in forest trees. Biol. Res. 40, 291–297. 10.4067/S0716-97602007000400003 18449457

[B38] Loake G. Grant M. (2007). Salicylic acid in plant defence -the players and protagonists. Curr. Opin. Plant Biol. 10, 466–472. 10.1016/j.pbi.2007.08.008 17904410

[B39] Ma W. Berkowitz G. A. (2007). The grateful dead: calcium and cell death in plant innate immunity. Cell. Microbiol. 9, 2571–2585. 10.1111/j.1462-5822.2007.01031.x 17714518

[B40] Mahomed W. van den Berg N. (2011). EST sequencing and gene expression profiling of defence-related genes from *Persea americana* infected with *Phytophthora cinnamomi*. BMC Plant Biol. 11:167. 10.1186/1471-2229-11-167 22108245 PMC3233532

[B41] Majumdar N. Wessel T. Marks J. (2015). Digital PCR modeling for maximal sensitivity, dynamic range and measurement precision. PLoS ONE 10:e0118833. 10.1371/journal.pone.0118833 25806524 PMC4373789

[B42] Martins L. Castro J. Macedo W. Marques C. Abreu C. (2007). Assessment of the spread of chestnut ink disease using remote sensing and geostatistical methods. Eur. J. Plant Pathol. 119, 159–164. 10.1007/s10658-007-9155-3

[B43] Miyakawa T. Hatano K. Miyauchi Y. Suwa Y. Sawano Y. Tanokura M. (2014). A secreted protein with plant-specific cysteine-rich motif functions as a mannose-binding lectin that exhibits antifungal activity. Plant Physiol. 166, 766–778. 10.1104/pp.114.242636 25139159 PMC4213107

[B44] Mohr P. G. Cahill D. M. (2007). Suppression by ABA of salicylic acid and lignin accumulation and the expression of multiple genes, in *Arabidopsis* infected with *Pseudomonas syringae* pv. tomato. Funct. Integr. Genomics 7, 181–191. 10.1007/s10142-006-0041-4 17149585

[B45] Montesinos E. (2007). Antimicrobial peptides and plant disease control. FEMS Microbiol. Lett. 270, 1–11. 10.1111/j.1574-6968.2007.00683.x 17371298

[B46] Mur L. A. J. Kenton P. Lloyd A. J. Ougham H. Prats E. (2008). The hypersensitive response; the centenary is upon us but how much do we know? J. Exp. Bot. 59, 501–520. 10.1093/jxb/erm239 18079135

[B47] Oñate-Sánchez L. Singh K. B. (2002). Identification of *Arabidopsis* ethylene-responsive element binding factors with distinct induction kinetics after pathogen infection. Plant Physiol. 128, 1313–1322. 10.1104/pp.010862 11950980 PMC154259

[B48] Oßwald W. Fleischmann F. Rigling D. Coelho A. C. Cravador A. Diez J. (2014). Strategies of attack and defence in woody plant–*Phytophthora* interactions. For. Pathol. 44, 169–190. 10.1111/efp.12096

[B49] Pandey G. K. Grant J. J. Cheong Y. H. Kim B. G. Li L. Luan S. (2005). ABR1, an APETALA2-domain transcription factor that functions as a repressor of ABA response in *Arabidopsis*. Plant Physiol. 139, 1185–1193. 10.1104/pp.105.066324 16227468 PMC1283757

[B50] Pitzschke A. Schikora A. Hirt H. (2009). MAPK cascade signalling networks in plant defence. Curr. Opin. Plant Biol. 12, 421–426. 10.1016/j.pbi.2009.06.008 19608449

[B51] Redondo M. Á. Pérez-Sierra A. Abad-Campos P. Torres L. Solla A. Reig-Armiñana J. (2015). Histology of *Quercus ilex* roots during infection by *Phytophthora cinnamomi*. Trees 29, 1943–1957. 10.1007/s00468-015-1275-3

[B52] Robin C. Smith I. Hansen E. M. (2012). Phytophthora cinnamomi. For. Phytophthoras 2. 10.5399/osu/fp.2.1.304121724173

[B53] Salvi S. Casadio V. Conteduca V. Burgio S. L. Menna C. Bianchi E.. (2015). Circulating cell-free AR and CYP17A1 copy number variations may associate with outcome of metastatic castration-resistant prostate cancer patients treated with abiraterone. Br. J. Cancer 112, 1717–1724. 10.1038/bjc.2015.128 25897673 PMC4430717

[B54] Santos C. Machado H. Correia I. Gomes F. Gomes-Laranjo J. Costa R. (2015). Phenotyping *Castanea* hybrids for *Phytophthora cinnamomi* resistance. Plant Pathol. 64, 901–910. 10.1111/ppa.12313

[B55] Sawano Y. Miyakawa T. Hiroshi Y. Tanokura M. Hatano K. (2007). Purification, characterization, and molecular gene cloning of an antifungal protein from Ginkgo biloba seeds. Biol. Chem. 388, 273–280. 10.1515/BC.2007.030 17338634

[B56] Schoch G. A. Nikov G. N. Alworth W. L. Werck-Reichhart D. (2002). Chemical inactivation of the cinnamate 4-hydroxylase allows for the accumulation of salicylic acid in elicited cells. Plant Physiol. 130, 1022–1031. 10.1104/pp.004309 12376665 PMC166627

[B57] Sefrioui D. Sarafan-Vasseur N. Beaussire L. Baretti M. Gangloff A. Blanchard F.. (2015). Clinical value of chip-based digital-PCR platform for the detection of circulating DNA in metastatic colorectal cancer. Dig. Liver Dis. 47, 884–890. 10.1016/j.dld.2015.05.023 26160500

[B58] Serrazina S. Santos C. Machado H. Pesquita C. Vicentini R. Pais M. S. (2015). *Castanea* root transcriptome in response to *Phytophthora cinnamomi* challenge. Tree Genet. Genomes 11, 1–19. 10.1007/s11295-014-0829-7

[B59] Shimono M. Koga H. Akagi A. Hayashi N. Goto S. Sawada M.. (2012). Rice WRKY45 plays important roles in fungal and bacterial disease resistance. Mol. Plant Pathol. 13, 83–94. 10.1111/j.1364-3703.2011.00732.x 21726399 PMC6638719

[B60] Stabley D. L. Harris A. W. Holbrook J. Chubbs N. J. Lozo K. W. Crawford T. O.. (2015). SMN1 and SMN2 copy numbers in cell lines derived from patients with spinal muscular atrophy as measured by array digital PCR. Mol. Genet. Genomic Med. 3, 248–257. 10.1002/mgg3.141 26247043 PMC4521962

[B61] Stevanato P. Biscarini F. (2016). Digital PCR as new approach to SNP genotyping in sugar beet. Sugar Tech. 18, 429–432. 10.1007/s12355-015-0408-8

[B62] Tena G. Boudsocq M. Sheen J. (2011). Protein kinase signaling networks in plant innate immunity. Curr. Opin. Plant Biol. 14, 519–529. 10.1016/j.pbi.2011.05.006 21704551 PMC3191242

[B63] ten Hove C. A. Bochdanovits Z. Jansweijer V. M. A. Koning F. G. Berke L. Sanchez-Perez G. F.. (2011). Probing the roles of LRR RLK genes in *Arabidopsis thaliana* roots using a custom T-DNA insertion set. Plant Mol. Biol. 76, 69–83. 10.1007/s11103-011-9769-x 21431781 PMC3097349

[B64] van der Vossen E. Sikkema A. Hekkert B. te L. Gros J. Stevens P.. (2003). An ancient R gene from the wild potato species *Solanum bulbocastanum* confers broad-spectrum resistance to *Phytophthora infestans* in cultivated potato and tomato. Plant J. 36, 867–882. 10.1046/j.1365-313X.2003.01934.x 14675451

[B65] Vlot A. C. Dempsey D. A. Klessig D. F. (2009). Salicylic acid, a multifaceted hormone to combat disease. Annu. Rev. Phytopathol. 47, 177–206. 10.1146/annurev.phyto.050908.135202 19400653

[B66] Wang H. Ng T. B. (2000). Ginkbilobin, a novel antifungal protein from *Ginkgo biloba* seeds with sequence similarity to embryo-abundant protein. Biochem. Biophys. Res. Commun. 279, 407–411. 10.1006/bbrc.2000.3929 11118300

[B67] Wang X. Li Q. Niu X. Chen H. Xu L. Qi C. (2009). Characterization of a canola C2 domain gene that interacts with PG, an effector of the necrotrophic fungus *Sclerotinia sclerotiorum*. J. Exp. Bot. 60, 2613–2620. 10.1093/jxb/erp104 19407339 PMC2692008

[B68] Wiethölter N. Graessner B. Mierau M. Mort A. J. Moerschbacher B. M. (2003). Differences in the methyl ester distribution of homogalacturonans from near-isogenic wheat lines resistant and susceptible to the wheat stem rust fungus. Mol. Plant. Microbe. Interact. 16, 945–952. 10.1094/MPMI.2003.16.10.945 14558696

[B69] Wittstock U. Gershenzon J. (2002). Constitutive plant toxins and their role in defense against herbivores and pathogens. Curr. Opin. Plant Biol. 5, 300–307. 10.1016/S1369-5266(02)00264-9 12179963

[B70] Yang B. Jiang Y. Rahman M. H. Deyholos M. K. Kav N. N. V. (2009). Identification and expression analysis of WRKY transcription factor genes in canola (*Brassica napus* L.) in response to fungal pathogens and hormone treatments. BMC Plant Biol. 9:68. 10.1186/1471-2229-9-68 19493335 PMC2698848

[B71] Zentmyer G. A. (1988). Origin and distribution of four species of *Phytophthora*. Trans. Br. Mycol. Soc. 91, 367–378. 10.1016/S0007-1536(88)80111-6

[B72] Zhang J. Peng Y. Guo Z. (2008). Constitutive expression of pathogen-inducible OsWRKY31 enhances disease resistance and affects root growth and auxin response in transgenic rice plants. Cell Res. 18, 508–521. 10.1038/cr.2007.104 18071364

[B73] Zhang K. M. Zheng F. C. Li Y. D. Ann P. J. Ko W. H. (1994). Isolates of *Phytophthora-Colocasiae* from Hainan-Island in China - evidence suggesting an Asian origin of this species. Mycologia 86, 108–112. 10.2307/3760724

[B74] Zhang Y. Lubberstedt T. Xu M. (2013). The genetic and molecular basis of plant resistance to pathogens. J. Genet. Genomics 40, 23–35. 10.1016/j.jgg.2012.11.003 23357342

