# Supplementary figures and images for: Expression Profiling of Castanea Genes during Resistant and Susceptible Interactions with the Oomycete Pathogen Phytophthora cinnamomi Reveal Possible Mechanisms of Immunity
Source: Front Plant Sci. 2017 Apr 11;8:515. doi: 10.3389/fpls.2017.00515 (PMC5387079; doi:10.3389/fpls.2017.00515)

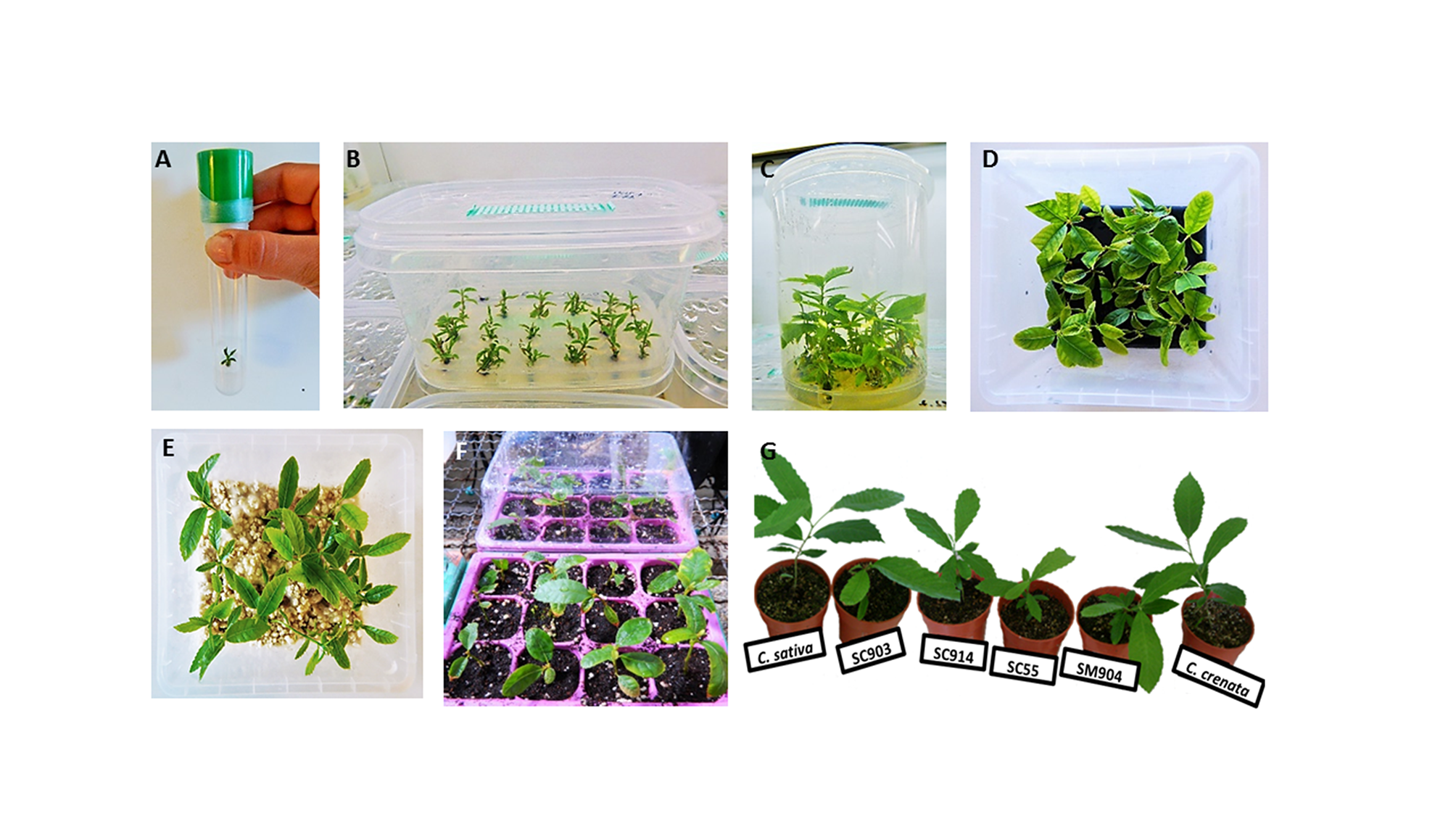

Supplement: Supplementary Figure 1 — Plant biological replicates of the different genotypes used in this study, obtained by in vitro culture. (A–D) In vitro micropropagation phases, (A) Establishment of an axillary node explant from the F1 mother plant; (B) Shoot multiplication; (C) Shoot elongation; (D) Pre-rooting in activated charcoal medium. (E–G) Ex-vitro phases; (E) Ex-vitro rooting, (F) Acclimatization, plantlets primary hardening; (G) Plantlets with 80 days after acclimatization used for inoculation. [file Image1.TIF]

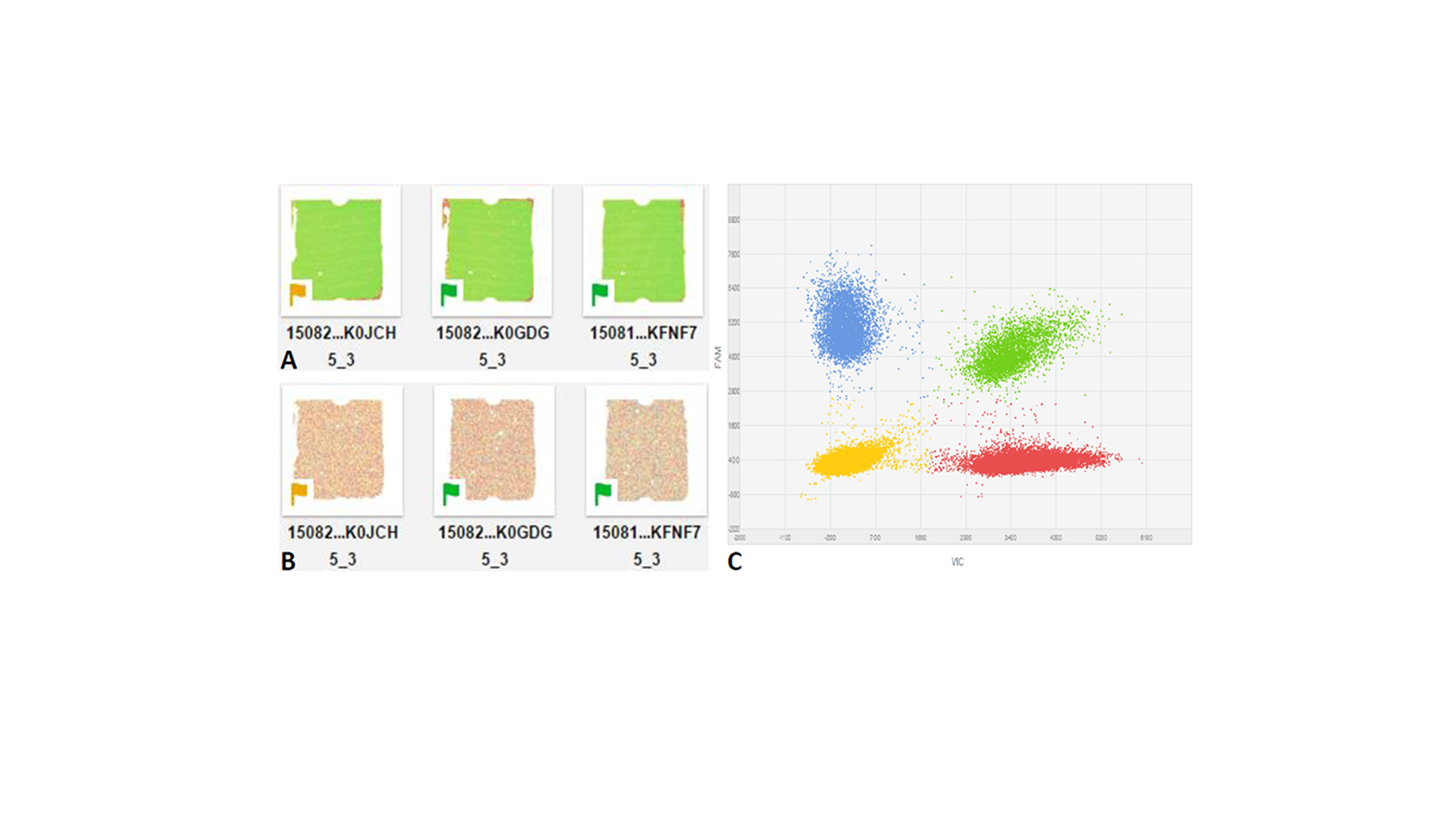

Supplement: Supplementary Figure 2 — Representative QS3D chip views and respective plot of Cast_WRKY 31 and Cast_Myb4 expression for three biological replicates (C. crenata, 48 hpi). (A) Chip views depicting color by quality. (B) Chip views depicting color by calls. (C) Scatter plot view from merging the three biological replicates. The data points on chip (B) and plot views (C) are color-coded according to the following fluorophores' color: FAM (blue), VIC (red), FAM + VIC (green) and not amplified (yellow). Relative intensities of FAM were plotted against VIC. [file Image2.TIF]
