# Supplementary material for: Expression Profiling of Castanea Genes during Resistant and Susceptible Interactions with the Oomycete Pathogen Phytophthora cinnamomi Reveal Possible Mechanisms of Immunity
Source: Front Plant Sci. 2017 Apr 11;8:515. doi: 10.3389/fpls.2017.00515 (PMC5387079; doi:10.3389/fpls.2017.00515)
Supplement: Supplementary Table 1 — Primers and probe sequences and fluorophores of eight Castanea crenata (Japanese, resistant) candidate genes to P. cinnamomi resistance. Contig names like in (Serrazina et al., 2015) [file Table1.DOCX]

Supplementary Table 1. Primers and probe sequences and fluorophores of eight *Castanea crenata* (Japanese, resistant) candidate genes to *P. cinnamomi* resistance. Contig names like in Serrazina et al., 2015.

| Gene acronyms | Contig name | Primer forward sequence | Primer reverse sequence | Probe Sequence |
| --- | --- | --- | --- | --- |
| *Cast_Gnk2-like* | CCI_CCN_005174 | CACCACGACAAAGAGCAAGT | CCACCAATGACCCATATGAA | ACCAAAGCCCAGGAGAGG |
| *Cast_PE-2* | CCI_CCN_002220 | TGACATCAACGGCAAGAGAT | AATGTCAAGTGCAGCCAAAC | CCAGGCCCGACACGTCTCAA |
| *Cast_ABR1* | CCI_CCN_001635 | GATGTGGAGTCTCCCTGTGA | TCTGCTCCTGCTTTTGCTT | CGCCCCCTCTTTTGGCCA |
| *Cast_C2CD* | CCI_CCN_008363 | CATGTGGAAGAGGAAACCT | GGAAAACTGAAATCAATTGAAG | ACGGTGGATGGAAACAGTCTGCA |
| *Cast_LRR-RLK* | CCI_CCN_000829 | CAATTCTCGAAAGTTGAACGA | GCTTAGGACTCACCCAATGC | TCACCGGCCAATCTGCAATTG |
| *Cast_Myb4* | CCI_CCN_004144 | TACAGCCCAATTTCCATTCA | CCAGCTCCAATGAAAAGGTT | TGGAACCAGACTATAGCGATGGCTCA |
| *Cast_WRKY 31* | CCI_CCN_000812 | GGTCTCTTCATCGGAAGGAA | ACAAGCCGCTCCTCACTAAT | ACGGTCAGGATCGCCCGGTA |
| *Cast_RNF5* | CCI_CCN_006887 | GGATTCCGTCAGCGTACAG | AGCAGCTCATGTTCCGATAG | TGAAGAGGCTGCTTTTGCTTATCGC |
